# The Relationship Between Serum Albumin Levels and Sepsis in Patients Admitted to a Tertiary Care Center in India

**DOI:** 10.7759/cureus.59424

**Published:** 2024-04-30

**Authors:** Hemanth G Kumar, Kirubhakaran Kanakaraju, Vaiera A. C. Manikandan, Vishal Patel, Chittimalla Pranay

**Affiliations:** 1 Internal Medicine, Vinayaka Mission's Kirupananda Variyar Medical College and Hospital, Salem, IND; 2 General Medicine, Vinayaka Mission's Kirupananda Variyar Medical College and Hospital, Salem, IND

**Keywords:** vasoconstrictor agents, systemic inflammatory response syndrome, mortality, intensive care units, sepsis, hypoalbuminemia

## Abstract

Introduction

Sepsis poses a significant threat in Indian hospitals, with high mortality rates and complications. This study explores the correlation between serum albumin levels and sepsis outcomes in an intensive care unit (ICU) setting. The challenges of diagnosing tropical infections further complicate sepsis management in India.

Methodology

A longitudinal study was conducted at Vinayaka Mission’s Kirupananda Variyar Medical College and Hospital, Salem, India. Adult patients admitted between July 2020 and March 2021 with sepsis were included. Serum albumin levels, demographic data, and clinical outcomes were analyzed. The study used a convenient sampling technique with a sample size of 102 patients.

Results

Among the 102 patients in the ICU, 22 have expired and the mortality rate in the study was 21.6%. Hypoalbuminemia was present in 56.9% (n = 58) of the patients. The mortality rate is higher among the sepsis patients with the occurrence of hypoalbuminemia (29.3%) compared to patients without hypoalbuminemia (11.4%) and the difference in proportion between the two groups was statistically significant (p-value = 0.029). The requirement of vasopressor support is higher among sepsis patients with the occurrence of hypoalbuminemia (56.9%) compared to patients without hypoalbuminemia (27.3%). The chi-square test reveals that the difference in proportion between the two groups was statistically significant (p-value = 0.005). No substantial impact on systemic inflammatory response scores, readmission to ICU, or progression to chronic illness was observed based on albumin levels.

Conclusion

This study underscores the predictive value of hypoalbuminemia in sepsis outcomes. Patients with decreased albumin levels showed higher mortality rates and increased vasopressor usage. While albumin levels did not significantly influence certain parameters, hypoalbuminemia may serve as an indicator of severity and adverse prognosis in sepsis, emphasizing the need for further research and tailored interventions.

## Introduction

Sepsis is a major threat in Indian hospitals, with nearly 60% of patients with severe sepsis not surviving past their initial hospitalization and a 57.6% death rate within 28 days [1]. In Indian intensive care units (ICUs), tropical infections further complicate sepsis, leading to multiple organ failure. These infections often present with common symptoms like fever and muscle pain (fever-myalgia), fever and joint pain (fever-arthralgia), fever and jaundice (fever-icterus), fever and rash, or acute encephalitis syndrome [2]. The challenges in diagnosing tropical infections stem from their varied manifestations, the involvement of multiple bodily systems, and the absence of established clinical diagnostic criteria. Compounding these difficulties are the limited sensitivity of testing methods and the high costs associated with, as well as the restricted availability of, isolation methods. These factors collectively contribute to the frequent misdiagnosis of such infections [3].

While extensive research on multiple organ dysfunction syndrome (MODS) in pediatric populations has been conducted in India, studies involving adults have predominantly centered on specific diagnoses like leptospirosis, dengue fever, and malaria. This has resulted in a notable gap in knowledge regarding the fundamental causes of sepsis in the adult population [2,3].

Turning attention to the United States, sepsis stands out as a considerable healthcare challenge, with more than 1.3 million treated cases in 2013. The economic repercussions are substantial, as sepsis holds the distinction of being the most expensive illness to treat, accounting for $23.7 billion in costs and comprising 6.2% of the total expenditures associated with hospitalizations [4]. The mortality rate for sepsis in the United States is approximated at 16%, although certain reports propose a potentially higher figure of 29.9% [5]. Importantly, individuals admitted to hospitals with sepsis encounter an eightfold higher risk of mortality and a 75% longer duration of hospitalization compared to patients with other medical conditions [5].

Despite the rising incidence of sepsis, the adoption of bundled treatment strategies, in alignment with the recommendations of the Surviving Sepsis Campaign, has played a role in reducing mortality rates over time. However, individuals who survive sepsis face enduring complications, encompassing multiple organ dysfunction or failure, myopathy, mental health challenges, and an increased risk of mortality in the months and years following the sepsis episode [6,7]. These intricate challenges emphasize the pressing necessity for thorough research and healthcare strategies aimed at addressing sepsis on a global scale.

Against the background delineated above, the current investigation endeavors to assess the connection between serum albumin levels at admission and their implications for patient outcomes in sepsis within the ICU. This study aims to explore the intricate relationship between initial serum albumin concentrations and the subsequent clinical prognosis of individuals admitted with sepsis, contributing to a nuanced understanding of the factors influencing outcomes in this critical healthcare scenario. By evaluating serum albumin levels at the point of admission, the research aims to illuminate the potential significance of this biomarker in predicting and influencing the trajectory of sepsis cases within the ICU setting. The findings from this study aspire to inform healthcare strategies and interventions tailored to enhance outcomes for septic patients in the ICU.

## Materials and methods

Research design

This study employs a longitudinal study to investigate the association between admission serum albumin levels and negative outcomes of adults admitted to ICU with sepsis. The research design aims to explore relationships with in-hospital mortality, ICU length of stay, progression to chronic critical illness, vasopressor usage, and ICU readmission.

Source of data and study duration

The collection of data occurs within the Medical and Surgical ICU, which comprises 20 beds, at Vinayaka Mission’s Kirupananda Variyar Medical College and Hospital in Salem, Tamil Nadu. Adult patients admitted between July 2020 and March 2021 with a primary diagnosis of sepsis were included in the study. The examination of serum albumin levels upon admission was subsequently accompanied by the monitoring of patient outcomes until discharge or death.

Inclusion and exclusion criteria

Inclusion criteria involve adult patients of all genders aged over 18 years, presenting with signs and symptoms of sepsis. The adult patients were diagnosed with sepsis if they satisfied at least two of the clinical criteria under the bedside clinical score termed quick SOFA (qSOFA). The clinical criteria are alteration in mental status, systolic blood pressure ≤ 100 mmHg, or respiratory rate ≥ 22/min. Exclusion criteria encompass severe hepatic dysfunction, nephrotic syndrome, undrained surgical sepsis sources, people living with HIV/AIDS or pregnancy, administration of human albumin within three weeks prior to severe sepsis onset, pre-existing cardiac arrest, death within 24 hours, or do-not-resuscitate orders.

Sampling technique and sample size

This study employed a convenient sampling technique, wherein all patients admitted to the ICU were recruited as study participants. A meta-analysis conducted by Bauer et al. in 2020, encompassing 170 articles assessing mortality in sepsis, revealed a 30-day mortality rate of 34.7% among patients with sepsis [8]. Considering this prevalence, with a 10% absolute error, a 95% confidence interval, and using the formula 3.84 * p * q / d^2^, the calculated minimum sample size required was 92. In our study, 102 samples were recorded.

Sample collection methods

Serum albumin levels considered a predictor variable, are collected using standard hospital procedures, with a reference range of 3.5-5.5 g/dl. Registered nurses in the ICU collected specimens, adhering to protocols accredited by the College of American Pathologists. The analysis includes all albumin values and the respective collection times (in hours).

Demographic and dependent variables

Demographic variables include age, gender, and comorbidities (type 2 diabetes, systemic hypertension, coronary artery disease (CAD), chronic kidney disease (CKD), and cerebrovascular accident (CVA)). Dependent variables comprise mortality, ICU length of stay, progression to chronic critical illness, vasopressor use, and ICU readmission. To meet the criteria for a systemic inflammatory response score (SIRS), it is required that at least two of the criteria are satisfied [9]. 1) If the body temperature exceeds 38⁰C or falls below 36⁰C; 2) if the heart rate is higher than 90 beats per minute; 3) if the respiratory rate exceeds 20 breaths per minute or the partial pressure of CO_2_ drops below 32 mm Hg; 4) if the leukocyte count is greater than 12000 or less than 4000 per microliter, or if there is an occurrence of over 10% immature forms or bands.

Ethical consideration

Approval for conducting this study was obtained through the ethical clearance process of the Institutional Ethics Committee of Kirupananda Variyar Medical College and Hospital (Approval No. VMKVMC&H/IEC/20/20). Following the approval from the Institutional Ethics Committee, data was gathered from patients who met the inclusion criteria and study protocol, having provided their informed consent through a signed document.

Data analysis

The data analysis was carried out by employing IBM SPSS Statistics for Windows, Version 22 (Released 2013; IBM Corp., Armonk, New York, United States), and tables were generated using Microsoft Excel (Microsoft® Corp., Redmond, United States). Descriptive statistics, such as mean and standard deviation, were used for continuous variables like age, albumin levels, and duration of ICU stay. Frequency and percentage were employed for categorical variables like gender, comorbidities, and outcome because of sepsis. Using the chi-square test, we investigated the relationship between hypoalbuminemia and the occurrence of outcome variables because of sepsis.

## Results

A total of 253 patients are admitted to medical and surgical ICUs from July 2020 to March 2021. Out of that total, only 134 patients were fulfilling SIRS criteria. Of those 134 patients, 32 are excluded as they match the exclusion criteria.

In the present study, the mean age of the study samples was 52.06 ± 14.15. Among 102 patients, 62 (60.8%) males are diagnosed with sepsis. Half of the patients (50%) were admitted to the ICU for 1 to 5 days and one-fourth of the patients (26.5%) stayed in the ICU for 6 to 10 days. Serum albumin level was measured within 24 hours of admission of the patients. The mean serum albumin level of the patients was 3.29 ± 0.63 g/dl. Hypoalbuminemia was present in 56.9% of the patients, which suggests hypoalbuminemia was commonly present with sepsis, as it is a negative acute phase reactant. Among the 102 patients in the ICU, 22 had expired and the mortality rate in the study was 21.6%. Diabetes mellitus was the most common comorbidity present in the patients diagnosed with sepsis, which is present among 48% of the population. Systemic hypertension (10.8%) was the second most common comorbidity present, followed by CAD (9.8%), CKD (7.8%), and CVA (3,9%) in the descending order. Nearly 44% of the patients required vasopressor support during their ICU stays. Characteristics of the patients diagnosed with sepsis and admitted to ICU are shown in Table 1.

**Table 1:** Characteristics of the patients diagnosed with sepsis ICU: intensive care unit

Variables		Frequency	Percent
Agen in years		52.06 ± 14.154	
Gender	Female	40	39.2
	Male	62	60.8
Number of days admitted in ICU	1 to 5	51	50
	6 to 10	27	26.5
	11 to 15	13	12.7
	more than 15	11	10.8
Hypoalbuminemia	Yes	58	56.9
	No	44	43.1
Diabetes mellitus	Yes	49	48
	No	53	52
Systemic hypertension	Yes	11	10.8
	No	91	89.2
Coronary artery disease	Yes	10	9.8
	No	92	90.2
Chronic kidney disease	Yes	8	7.8
	No	94	92.2
Cerebro-vascular accident	Yes	4	3.9
	No	98	96.1
Vasopressor use	Yes	45	44.1
	No	57	55.9
Mortality	Yes	22	21.6
	No	80	78.4

All SIRS criterion components have a frequency greater than 50 in this study. Elevated white cell count is present in 82.4% of the population. Temperature <36⁰C and total count <4000 were not found in any patients from the study. Out of 102 patients, 48% of the patients matched three out of the four criteria of SIRS criteria. About 21.6% of patients matched all four criteria and 30.4% matched two criteria of the SIRS criteria. Table 2 describes the distribution of patients based on SIRS criteria.

**Table 2:** Distribution of patients based on SIRS criteria SIRS: systemic inflammatory response score

SIRS Criteria		Frequency	Percent
Temperature > 38⁰C	No	36	35.3
	Yes	66	64.7
Respiratory rate > 22/min	No	28	27.5
	Yes	74	72.5
Heart rate > 90/min	No	29	28.4
	Yes	73	71.6
Total count > 12000	No	18	17.6
	Yes	84	82.4
SIRS score	2	31	30.4
	3	49	48.0
	4	22	21.6

Out of 80 patients, 15% of the patients required readmission to the ICU within one month of discharge or shifted back to ICU from wards during the study period. Nearly 17.5% of the patients have progressed to chronic critical illness while 82.5% did not in this study. The distribution of the study participants according to their status of readmission to ICU and progression to chronic illness are shown in Table 3. Among 102 participants, 22 have expired in the ICU and the remaining 80 have been considered.

**Table 3:** Distribution of the study participants according to their status of readmission to ICU and progression to chronic illness (n = 80) ICU: intensive care unit

Variables		Frequency	Percent
Readmission to ICU	Yes	12	15
	No	68	85
Progression to chronic critical illness	Yes	14	17.5
	No	66	82.5

The number of patients with an SIRS score of 4 is found to be higher with the occurrence of hypoalbuminemia (25.9%) compared to patients without hypoalbuminemia (15.9%). The chi-square test reveals that the difference in proportion between the two groups was not statistically significant (p-value = 0.459). Hence, the SIRS score was not influenced by the existence of hypoalbuminemia in this study. The association between hypoalbuminemia and SIRS score among the study participants is shown in Table 4.

**Table 4:** Association between hypoalbuminemia and SIRS score among the study participants (n = 102) SIRS: systemic inflammatory response score

Hypoalbuminemia		SIRS score			Total	Chi-square value	p-value
		2	3	4			
Yes	Frequency	16	27	15	58	1.559	0.459
	Percent	27.6%	46.6%	25.9%	100%		
No	Frequency	15	22	7	44		
	Percent	34.1%	50.0%	15.9%	100%		

The requirement of vasopressor support is higher among sepsis patients with the occurrence of hypoalbuminemia (56.9%) compared to patients without hypoalbuminemia (27.3%). The chi-square test reveals that the difference in proportion between the two groups was statistically significant (p-value = 0.005). The association between hypoalbuminemia and vasopressor use among the study participants is shown in Table 5.

**Table 5:** Association between hypoalbuminemia and vasopressor use among the study participants (n = 102)

Hypoalbuminemia		Vasopressor use		Total	Chi-square value	p-value
		Yes	No			
Yes	Frequency	33	25	58	8.906	0.005
	Percent	56.90%	43.10%	100.00%		
No	Frequency	12	32	44		
	Percent	27.30%	72.70%	100.00%		

The mortality rate is higher among sepsis patients with the occurrence of hypoalbuminemia (29.3%) compared to patients without hypoalbuminemia (11.4%). The chi-square test reveals that the difference in proportion between the two groups was statistically significant (p-value = 0.029). The association between hypoalbuminemia and the number of mortalities among the study participants is shown in Table 6.

**Table 6:** Association between hypoalbuminemia and the number of mortalities among the study participants (n = 102)

Hypoalbuminemia		Mortality		Total	Chi-square value	p-value
		Yes	No			
Yes	Frequency	17	41	44	4.765	0.029
	Percent	29.30%	70.70%	100%		
No	Frequency	5	39	58		
	Percent	11.40%	88.60%	100%		

Readmission to ICU is lower among sepsis patients with the occurrence of hypoalbuminemia (9.8%) compared to patients without hypoalbuminemia (20.5%). The chi-square test reveals that the difference in proportion between the two groups was not statistically significant (p-value = 0.178). Hence, readmission to ICU among patients with sepsis was not influenced by the existence of hypoalbuminemia. The association between hypoalbuminemia and readmission to the ICU among the study participants is shown in Table 7.

**Table 7:** Association between hypoalbuminemia and readmission to ICU (excluding those who had died) among the study participants (n = 80) ICU: intensive care unit

Hypoalbuminemia		Readmission to ICU		Total	Chi-square value	p-value
		Yes	No			
Yes	Frequency	4	37	41	1.814	0.178
	Percent	9.80%	90.20%	100%		
No	Frequency	8	31	39		
	Percent	20.50%	79.50%	100%		

Progression to chronic critical illness is higher among sepsis patients with the occurrence of hypoalbuminemia (19.5%) compared to patients without hypoalbuminemia (15.4%). The chi-square test reveals that the difference in proportion between the two groups was not statistically significant (p-value = 0.627). Hence, progression to chronic critical illness among patients with sepsis was not influenced by the presence of hypoalbuminemia. The association between hypoalbuminemia and progression to chronic critical illness among the study participants is shown in Table 8.

**Table 8:** Association between hypoalbuminemia and progression to chronic critical illness (excluding those who had died) among the study participants (n = 80)

Hypoalbuminemia		Progression to chronic critical illness		Total	Chi-square value	p-value
		Yes	No			
Yes	Frequency	8	33	39	0.236	0.627
	Percent	19.50%	80.50%	100%		
No	Frequency	6	33	41		
	Percent	15.40%	84.60%	100%		

## Discussion

Albumin has been recognized for its partial anti-inflammatory effects and its potential benefits for patients experiencing sepsis [10]. In cases of sepsis, individuals are prone to exhibit reduced levels of serum albumin, and this is strongly linked to an unfavorable prognosis. Among the 102 patients admitted to the ICU in this study, the mortality rate was found to be 21.6%. This mortality rate is lower when compared to the study conducted by Todi et al. in India in 2010, where the mortality rate because of sepsis was reported to be 59.2% [1].

In this study, the mortality rate was higher among sepsis patients with hypoalbuminemia (29.3%) compared to those without hypoalbuminemia (11.4%). The serum albumin levels were measured within 24 hours of admission, and the mean serum albumin level for the patients was 3.29 ± 0.63 g/dl. A study by Kendall et al. in sepsis revealed that when admission serum albumin was ≤2.45 g/dl and the lowest serum albumin during hospitalization was ≤1.45 g/dl, the probability of survival in the hospital declined by 70.6% and 76.4%, respectively [11]. Arnau-Barrés et al. conducted a study on elderly sepsis patients, revealing that an albumin level <2.6 g/dl was identified as a prognostic factor with 30-day mortality, even after adjusting for age, gender, and comorbidities [12]. In septic and septic shock patients admitted to the emergency department, the initial albumin level was found to be the most significant contributor to clinical outcomes [13]. A recent retrospective study conducted by Lee et al. in 2017, involving 725 sepsis patients, revealed that a reduced level of serum albumin (<2.5 g/dl) was associated with one-year mortality, with an odds ratio of 2.69 [14].

Our study findings showed a significant association between decreased levels of albumin and poor prognosis in sepsis. Several potential mechanisms might explain this phenomenon. First, severe oxidative stress and capillary leak have been correlated with lower serum albumin levels in sepsis. This aligns with the dysregulated host response that is central to the pathophysiology of sepsis and contributes to fatal outcomes [15,16]. Second, from a physiological perspective, albumin is synthesized in the liver, and the impairment of liver function in sepsis may cause a deficiency in albumin synthesis [17]. Third, inflammation damages renal function, leading to proteinuria through the upregulation of glomerular infiltration and causing the leakage of albumin [18]. In sepsis, gastrointestinal function is typically impaired, partially affecting the absorption of nutrients and resulting in malnutrition status [19].

In the current study, the need for vasopressor support is more pronounced among sepsis patients with hypoalbuminemia (56.9%) compared to those without hypoalbuminemia (27.3%). Previous literature has also discussed the association between hypoalbuminemia and the requirement for vasopressor support. In the management of refractory hypotension in sepsis, norepinephrine is the preferred vasopressor [20]. Dilation of blood vessels and compromised capillary integrity are associated with elevated levels of oxidative stress, heightening the likelihood of vasopressor utilization. Symptoms such as hypotension, vasodilation, and inadequate tissue perfusion signify an aberrant host response in sepsis. The requirement for vasopressors may show an overly aggressive host reaction [6]. This investigation established a connection between diminished serum albumin levels and the utilization of vasopressors.

Among the 80 patients in this study, 15% caused readmission to the ICU within one month of discharge or were transferred back to the ICU from wards during the study period. This result can be juxtaposed with the study conducted by Lai et al. in Taiwan in 2006, where a readmission rate of 13.13% was observed among 192,201 patients admitted to the ICU, and the follow-up period extended beyond 200 days [21].

Limitations

The generalizability of these findings is limited because of the homogeneity of the study population's demographics and the reliance on data from a single collection facility. Enhancing generalizability could be achieved through a larger sample size drawn from diverse institutions with nationally representative demographic characteristics. While serum albumin levels were only measured at admission in this study, conducting serial monitoring throughout the hospital stay could provide a more comprehensive understanding of sepsis prognosis. Future research could benefit from a multi-centered cohort study, investigating the optimal frequency for repeated serum albumin level assessments and developing predictive models for ICU mortality. The exclusion of data on subclinical diseases, such as subclinical nonalcoholic fatty liver disease, may have implications for a more comprehensive interpretation of the study's results.

## Conclusions

This study highlights the predictive importance of hypoalbuminemia in sepsis within the ICU context. Patients with decreased albumin levels exhibited elevated mortality rates and a higher likelihood of vasopressor usage compared to those with normal albumin levels. The study did not find a significant impact of albumin levels on SIRS scores. There was no statistically significant influence on readmission to the ICU or progression to chronic critical illness based on albumin levels. These results show hypoalbuminemia may serve as a potential indicator of severity and an adverse prognosis in sepsis. Further investigations are necessary to delve into the underlying mechanisms and assess the clinical implications for managing sepsis in critically ill patients.
